# Growth hormone supplementation ameliorates blastocyst euploidy rates and improves pregnancy outcomes in women undergoing preimplantation genetic testing for aneuploidy cycles

**DOI:** 10.3389/fendo.2023.1117706

**Published:** 2023-03-06

**Authors:** Qingqing Guo, Peihao Liu, Wei Zhou, Mingdi Xia, Jing Li, Juanjuan Lu, Jin-Long Ma, Zi-Jiang Chen, Junhao Yan

**Affiliations:** ^1^ Center for Reproductive Medicine, Shandong University, Jinan, Shandong, China; ^2^ Key Laboratory of Reproductive Endocrinology of Ministry of Education, Shandong University, Jinan, Shandong, China; ^3^ Shandong Key Laboratory of Reproductive Medicine, Jinan, Shandong, China; ^4^ Shandong Provincial Clinical Research Center for Reproductive Health, Jinan, Shandong, China; ^5^ Shandong Technology Innovation Center for Reproductive Health, Jinan, Shandong, China; ^6^ National Research Center for Assisted Reproductive Technology and Reproductive Genetics, Shandong University, Jinan, Shandong, China; ^7^ Shanghai Key Laboratory for Assisted Reproduction and Reproductive Genetics, Shanghai, China; ^8^ Center for Reproductive Medicine, Ren Ji Hospital, School of Medicine, Shanghai Jiao Tong University, Shanghai, China

**Keywords:** growth hormone, preimplantation genetic testing, aneuploidy, blastocyst, frozen embryo transfer

## Abstract

**Background:**

Growth hormone (GH) supplementation has been shown to improve oocyte quality and live birth, but few studies have examined whether GH can reduce embryonic aneuploidy. Chromosomal abnormalities in preimplantation embryos have been regarded as the principal cause of implantation failure and miscarriage, and an increased percentage of aneuploid embryos has been observed in patient cohorts with unexplained recurrent pregnancy loss (RPL), recurrent implantation failure (RIF), and advanced maternal age.

**Methods:**

This prospective cohort study was conducted on women whose previous PGT-A cycle ended up with no transferrable blastocysts, or the aneuploidy rate was above 50% and no live birth was acquired. The participants were divided into GH co-treatment and comparison groups according to whether GH was administered in the subsequent PGT-A cycle. In addition, within the GH co-treatment group, the previous failed cycle constituted the self-control group.

**Results:**

208 women were recruited in the study (GH co-treatment group: 96 women, comparison group: 112 women). Compared to the self-control and comparison groups, the rate of euploid blastocysts was significantly higher in the GH co-treatment group (GH vs self-control: 32.00% vs 9.14%, odds ratio [OR]: 4.765, 95% confidence interval [CI]: 2.420–9.385, P < 0.01; GH vs comparison: 32.00% vs. 21.05%, OR: 1.930, 95% CI: 1.106–3.366, P = 0.021), and their frozen embryo transfers resulted in more pregnancies and live births. In the subgroup analysis, for the <35 and 35-40 years groups, the euploidy rate in the GH co-treatment group was significantly higher than those in the self-control and comparison groups, but in the >40 years group, there was no difference in euploidy rate.

**Conclusion:**

Our study presents preliminary evidence that GH supplementation may ameliorate blastocyst aneuploidy and improve pregnancy outcomes in women who have previously experienced pregnancy failures along with high aneuploidy rates, particularly in those younger than 40 years. Therefore, the use of GH in such women should be considered. However, considering the limited sample size and mixed indications for PGT-A, further scientific research on the underlying mechanism as well as clinical trials with larger sample sizes are needed to confirm the effects and optimal protocols.

## Introduction

The prognosis of *in vitro* fertilization (IVF) depends largely on the quality of the generated oocytes and embryos. Currently, numerous strategies are being applied to enhance oocyte quality and produce high-quality embryos to improve overall IVF outcomes. Over the past few decades, several investigators have aimed to prove that growth hormone (GH) supplementation during IVF cycles improves IVF outcomes; specifically, some researchers have shown that human oocytes and cumulus cells have GH receptors (GHRs), allowing GH to exert a direct effect on follicular growth (1, 2). In addition, GH may also have an indirect effect on development of follicles by activating the synthesis of insulin-like growth factor-I (3).

Through animal experiments, researchers have shown that GH supplementation improves oocyte quality in aged mice by enhancing mitochondrial function (4). Another study showed that GH directly improves oocyte quality by upregulating GHRs and enhancing mitochondrial activity (5). In addition, GH co-treatment during IVF in older women has been shown to modulate the density of receptors in granulosa cells, which can improve luteinization and pregnancy outcomes in older patients (6). A recent systematic review of the effects of GH on endometrial improvement in women undergoing IVF showed that GH may improve IVF outcomes, especially in women with a thin endometrium; however, the quality of evidence was not high (7). Nonetheless, many studies have established that compared with untreated women, pregnancy, implantation, and live birth rates are augmented by exogenous GH supplementation in treated women (8–12). However, the role of GH supplementation in IVF is yet to be determined. One randomized controlled trial with poor responders was not able to identify any impact of GH supplementation on pregnancy outcomes (13), whereas a recent systematic review and meta-analysis showed that GH supplementation could improve certain reproductive outcomes for poor responders, with the exception of live birth rate (14). However, another meta-analysis revealed that GH supplementation improved live birth rate for poor ovarian responders (15). Thus, as findings on the application and benefits of GH adjuvant therapy for IVF differ, further research is required to determine whether GH supplementation truly improves the pregnancy outcomes for IVF patients.

Chromosomal abnormalities in preimplantation embryos have been regarded as the principal cause of implantation failure and miscarriage, and such abnormalities often result from advanced maternal age. An increased percentage of aneuploid embryos has been observed in patient cohorts with unexplained recurrent pregnancy loss (RPL), recurrent implantation failure (RIF), and advanced maternal age (16–19). Although GH supplementation does appear to improve oocyte quality, few studies have examined whether GH can reduce embryonic aneuploidy, and only one previous retrospective cohort study has explored the effect of GH supplementation on oocyte and embryo outcomes in IVF and preimplantation genetic testing for aneuploidy (PGT-A) cycles (20). To our knowledge, this is the first prospective study to assess the effects of GH administration on blastocyst aneuploidy in women who underwent PGT-A cycles.

## Materials and methods

### Experimental design

This was a prospective cohort study conducted among women whose previous PGT-A cycle ended up with no transferrable blastocysts, or the aneuploidy rate was above 50% and no live birth was acquired.

### Patient recruitment

We recruited 230 couples whose previous PGT-A cycles had not resulted in a live birth and who underwent a subsequent PGT-A cycle from January 2018 to September 2020 at the Center for Reproductive Medicine, Shandong University. The following patients were included: 1) women aged 21–45 years who underwent PGT-A for RPL or RIF or were of advanced maternal age, 2) women who experienced prior pregnancy failures with aneuploidy rates higher than 50%, and 3) women with no previous record of GH usage. The following patients were excluded: 1) women who underwent PGT for structural rearrangements or monogenic disorders; 2) women with known uterine or endometrial pathologies, such as uterine malformation, intrauterine adhesions, hydrosalpinx, endometriosis and adenomyosis; and 3) women with contraindications to GH.

The participants were divided into GH co-treatment and comparison groups based on GH administration. Two study groups were formed based on patient willingness to undergo GH supplementation. In addition, in the GH co-treatment group, the previous failed cycle constituted a self-control group.

### Ethical approval

All participant data were collected after obtaining informed consent from all patients. This study was approved by the Institutional Review Board for Reproductive Medicine at Shandong University, and was conducted according to the Declaration of Helsinki for Medical Research Involving Human Subjects.

### Clinical procedures

Appropriate ovarian stimulation protocols were administered based on individual patient ovarian reserve functions and prior ovarian stimulation protocols. Patients underwent ovarian stimulation *via* recombinant follicle-stimulating hormone (FSH) or human menopausal gonadotropin, with the initial dose determined based on the patient’s age, antral follicle count, and anti-Müllerian hormone (AMH) concentration. Human chorionic gonadotropin (HCG) (Pregnyl, Livzon, Guangdong, China) was administered to trigger final oocyte maturation when at least three follicles or dominant follicles attained a diameter of 18 mm. Approximately 36–38 h following HCG injection, transvaginal ultrasound-guided oocyte retrieval was performed. All oocytes from the patients underwent intracytoplasmic sperm injection.

During the GH supplementation cycles, GH (Saizen, Kinsey, Changchun, China) was administered subcutaneously daily at a dose of 2 IU. GH treatment was initiated on days 1–3 of the last menstrual period onset and lasting until the day of HCG injection, for a total of approximately 42 days. The comparison group received similar ovarian stimulation and other treatment protocols without GH supplementation.

### Biopsy procedure, next-generation sequencing protocol, and classification of the results

After fertilization, all embryos were cultured through the blastocyst stage. Following the Gardner scoring system (21), we selected good-quality blastocysts with a score of 4 BC or better on days 5 or 6 of embryo culture for trophectoderm biopsy using the laser method.

The biopsied trophectoderm cells were lysed and genomic DNA was amplified using whole genome amplification (WGA, SurePlex, Illumina, Inc., San Diego, CA, USA). The WGA products were then purified, quantified, and fragmented into libraries (100–200 bp). DNA fragments with unique adapter sequences were added for high-throughput analysis. After the libraries were sequenced by single-end dual indexing using NGS platforms (Illumina MiSeq), the reads were aligned with the human genome hg19 and filtered by deleting unmapped reads, duplicate reads, and reads with low mapping scores. Each chromosome was then divided into several intervals covering a 1 Mb sequence, and the filtered reads were mapped to the corresponding chromosome intervals or bins. Considering that the median autosomal read count was taken from copy number 2, if the generated plot showed copy numbers ranging from 1.7 to 2.3, the embryos were diagnosed as normal or euploid. Embryos were then diagnosed as abnormal or aneuploid if the median chromosomal copy number deviated from the default copy number (copy number <1.3 or >2.7), and diagnosed as mosaic when the copy number was between 2.3 and 2.7 or 1.3 and 1.7. Embryos were considered to have an unknown result if amplification failure occurred. However, the NGS detecting system cannot detect polyploidy.

### Single-embryo transfer

All embryos were cryopreserved and embryo transfer was performed after the second menstruation following oocyte retrieval. Based on the menstrual cycle of the patients, the endometrium was prepared through a natural ovulatory cycle or an artificial regimen. The luteal phase was supported with oral dydrogesterone twice daily (20 mg; Duphaston, Abbott, USA) and vaginal progesterone capsules once daily (200 mg; Utrogestan, Besins Manufacturing, Belgium). Single euploid blastocyst transfer was performed as recommended.

### Comparison and statistical analysis

The primary outcome measure was the rate of euploid blastocyst formation; the secondary outcomes were clinical pregnancy, miscarriage, and live birth rates after frozen-embryo transfer (FET). Clinical pregnancy was determined when a gestational sac was detected using transvaginal ultrasonography. Miscarriage was defined as pregnancy loss after detection of a gestational sac. At least one fetus was born alive after 28 weeks of pregnancy, which was defined as a live birth.

Quantitative variables are represented as mean and standard deviation, and qualitative variables as numbers and percentages. Quantitative variables were compared using the paired t-test, independent sample t-test, Wilcoxon signed-rank test, or non-parametric Mann–Whitney U test as appropriate. Qualitative variables were analyzed using the McNamar’s test, chi-square test, or Fisher’s exact test for expected frequencies < 5. Generalized estimating equations were used to compare the PGT-A analyses between the GH co-treatment and self-control groups, and the euploidy rate, clinical pregnancy rate, and live birth rate per transfer between the GH co-treatment and comparison groups. Binary Logistic regression was conducted to compare Live birth rate per group between the GH co-treatment and comparison groups. The candidate confounders for the blastocyst euploidy rate were ovarian stimulation protocol, total gonadotropin (Gn), and number of oocytes in the comparison between the GH co-treatment and self-control groups. Besides the above confounders, age, AMH, body mass index (BMI), infertility duration, infertility type, and the indication for PGT-A were also adjusted between the GH co-treatment and comparison groups. For pregnancy outcomes, with the exception of Live birth rate per group, the candidate confounders were age, BMI, endometrial thickness, endometrial preparation protocol, and indication for PGT-A. The candidate confounders for the Live birth rate per group were age, BMI, indication for PGT-A, and ovarian stimulation protocol.

Statistical analyses were performed using SPSS version 20.0 (IBM Corp, Armonk, NY, USA), and a two-tailed P < 0.05 was considered statistically significant.

## Results

**Figure 1:**
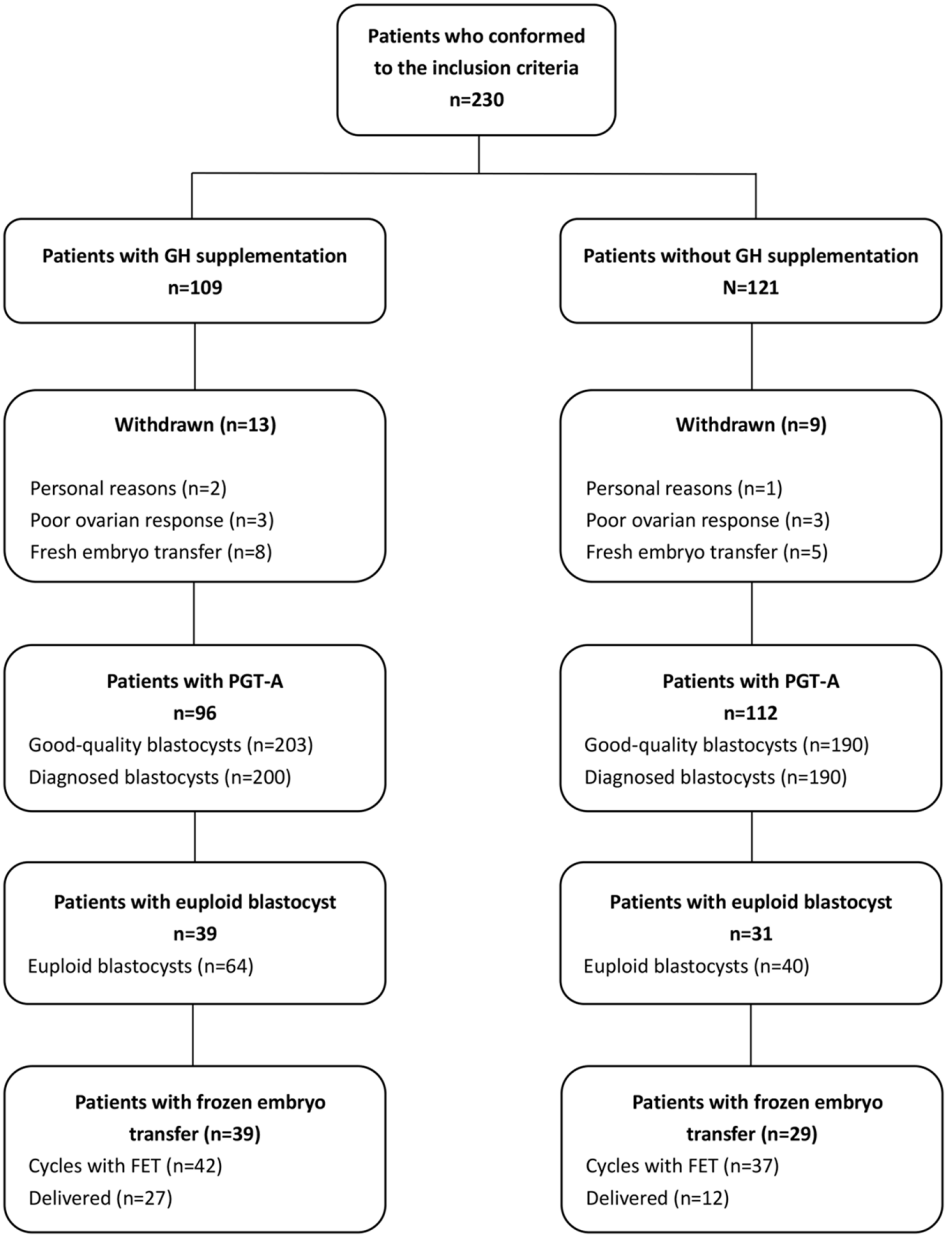
Diagrammatic representation showing the processes of patient inclusion and exclusion in the study.

Totals of 109 and 121 couples were enrolled in the GH co-treatment and comparison groups, respectively. Of them, eight patients in the GH co-treatment group and five in the comparison group refused PGT-A analysis and insisted on the transfer of fresh embryos. Three patients in each group cancelled oocyte retrieval because of poor ovarian response. Additionally, two patients in the GH co-treatment group and one in the comparison group withdrew from the study for personal reasons. Ultimately, 208 couples were enrolled in our study, with 96 in the GH co-treatment group and 112 in the comparison group (**Figure 1**). No adverse side effects were reported by any of the participants with GH supplementation, and nor were there any cases of ovarian hyperstimulation syndrome.

We did not observe any significant differences in patient demographic characteristics between the GH co-treatment and comparison groups. **Table 1** shows the age and BMI, as well as the AMH, FSH, luteinizing hormone, and estradiol (E2) concentrations of participants, proportions of various indications for PGT-A, and the proportion of patients with previous IVF cycles.

**Table 1:** Baseline demographic characteristics of patients.

	GH co-treatment group (n = 96)	Comparison group (n = 112)	*P* value
Age (years)	38.78 (4.13)	39.18 (3.76)	0.469
BMI (kg/m^2^)	23.81 (2.56)	23.98 (3.13)	0.667
AMH (ng/mL)	2.25 (2.71)	2.14 (2.07)	0.722
FSH (IU/L)	7.29 (2.16)	7.60 (2.72)	0.364
LH (IU/L)	4.62 (2.13)	5.01 (2.91)	0.287
Estradiol (pg/mL)	39.53 (19.83)	42.27 (20.98)	0.338
Infertility duration (years)	3.77 (4.17)	2.28 (2.45)	0.440
Primary infertility	8 (8.33%)	4 (3.57%)	0.142
Indication for PGT-A			
RPL	50 (52.08%)	54 (48.21%)	0.578
RIF	14 (14.58%)	14 (12.50%)	0.661
Advanced maternal age	32 (33.33%)	44 (39.29%)	0.374
Patients with previous IVF cycles	24 (25.00%)	25 (22.32%)	0.650

Data are presented as mean (standard deviation), N (%).

Comparisons were made using the independent sample t-test, non-parametric Mann–Whitney U test, or Pearson’s chi-squared test, as appropriate. Statistical significance was set at P < 0.05.

GH, growth hormone; BMI, body mass index; AMH, anti-Müllerian hormone; FSH, follicle-stimulating hormone; LH, luteinizing hormone; PGT-A, preimplantation genetic testing for aneuploidy; RPL, recurrent pregnancy loss; RIF, recurrent implantation failure; IVF, in vitro fertilization.

### Parameters during ovarian stimulation and embryonic indices

The parameters of ovarian stimulation and embryonic indices are summarized in **Table 2**. The cycle characteristics, namely, the duration of exogenous gonadotropin (Gn) treatment, total Gn dose, E2 concentration, and endometrial thickness on day of HCG administration, did not differ significantly between the GH co-treatment, comparison, and self-control groups. The number of oocytes retrieved, MII oocytes, and good-quality blastocysts in the GH co-treatment group tended to increase relative to the other two groups, but the difference was not statistically significant. The proportion of luteal phase ovarian stimulation was higher in the comparison group than that in the GH co-treatment group (7.14% vs 1.04%, *P*=0.040).

**Table 2:** Comparison of parameters during the ovarian stimulation and embryonic indices.

	GH co-treatment group (GH)	Self-control group (Non-GH)	*P* value^a^	Comparison group (Non-GH)	*P* value^b^
Number of enrolled patients	96	96		112	
Ovarian stimulation protocol					
GnRH agonist long	27 (28.13%)	28 (29.17%)	1.000	22 (19.64%)	0.151
GnRH agonist short	21 (21.88%)	29 (30.21%)	0.322	30 (26.79%)	0.412
GnRH antagonist	42 (43.75%)	30 (31.25%)	0.195	45 (40.18%)	0.603
Mild stimulation protocol	5 (5.21%)	7 (7.29%)	0.774	7 (6.25%)	0.748
Luteal phase ovarian stimulation	1 (1.04%)	2 (2.08%)	1.000	8 (7.14%)	0.040
Duration of Gn treatment (days)	10.13 (1.81)	10.38 (1.95)	0.358	9.88 (2.19)	0.376
Total Gn dose (IU)	2365.23 (908.75)	2230.73 (906.43)	0.170	2329.46 (905.90)	0.777
E2 on HCG day (pg/ml)	2547.48 (1841.98)	2704.63 (1626.9)	0.700	2373.91 (1519.07)	0.458
Endometrial thickness on HCG day (cm)	0.96 (0.25)	0.96 (0.26)	0.788	0.95 (0.22)	0.792
Number of oocytes	8.49 (6.81)	7.95 (4.46)	0.921	7.01 (4.67)	0.151
Number of MII oocytes	6.96 (5.66)	6.24 (3.95)	0.414	5.90 (4.09)	0.282
Number of 2PN	5.32 (4.31)	5.20 (3.07)	0.970	4.50 (3.08)	0.280
Number of good-quality cleaved embryos	2.71 (2.26)	3.01 (2.32)	0.260	2.62 (2.12)	0.806
Number of good-quality blastocysts	2.11 (1.85)	1.82 (1.58)	0.251	1.70 (1.72)	0.057
Good-quality blastocysts/number of 2PN	203/511 (39.73%)	175/499 (35.07%)	0.126	190/504 (37.70%)	0.507

Data are presented as mean (standard deviation), N, or n/N (%).

a Comparison between GH cotreatment and self-control groups.

b Comparison between the GH co-treatment and comparison groups.

The GH co-treatment and self-control groups were compared using the paired t-test, Wilcoxon signed-rank test, McNemar’s test, or Pearson’s chi-squared test, as appropriate. The GH co-treatment and comparison groups were compared using an independent sample t-test, non-parametric Mann–Whitney U test, Pearson’s chi-square test, or Fisher’s exact test, as appropriate. The rate of good-quality blastocysts was compared using Pearson’s chi-square test. Statistical significance was set at P < 0.05.

GH, growth hormone; GnRH, gonadotropin-releasing hormone; Gn, exogenous gonadotropin; E2, estradiol; HCG, human chorionic gonadotropin; 2PN, two pronuclear.

### PGT-A outcomes

In the GH co-treatment group, 200 of 203 blastocysts were diagnosed by NGS analysis, and in the self-control and comparison groups all blastocysts were diagnosed (175 blastocysts in the self-control group and 190 in the comparison group). The number of euploid blastocysts in the GH co-treatment group was higher than those in the self-control and comparison groups (GH vs self-control: 0.67 ± 0.97 vs 0.17 ± 0.45, *P* < 0.01; GH vs control: 0.67 ± 0.97 vs 0.36 ± 0.66, *P* = 0.02) (**Table 3**). In the adjusted analysis, the rate of euploid blastocysts was significantly higher in the GH co-treatment group than in the control and comparison groups (GH vs self-control: 32.00% vs 9.14%, odds ratio [OR]:4.765, 95% confidence interval [CI]:2.420–9.385, *P* < 0.01; GH vs. comparison: 32.00% vs 21.05%, OR: 1.930, 95% CI: 1.106–3.366, *P* = 0.021). In the subgroup analysis, for the <35 and 35-40 years groups, the euploidy rates in the GH co-treatment group were significantly higher than those in the self-control and comparison groups (<35 years: GH vs self-control: 60.98% vs 11.76%, *P* < 0.01; GH vs comparison: 60.98% vs 28.57%, *P*=0.003) (35-40 years: GH vs self-control: 34.31% vs 7.69%, *P* < 0.01; GH vs comparison: 34.31% vs 21.51%, P=0.047 but in the >40 years group, there was no difference in euploidy rates (**Table 3**). The numbers of patients with at least one euploid blastocyst were 39, 13, and 31 in the GH co-treatment, self-control group, and comparison group, respectively. Compared to the self-control group, the proportion of patients with euploid blastocysts was significantly higher in the GH co-treatment group (GH vs self-control: 40.63% vs. 13.54%, OR: 7.742, 95% CI: 2.950–19.926, *P* < 0.01); this proportion was also higher than that in the comparison group but the difference was not statistically significant (GH vs comparison: 40.63% vs 27.68%, OR: 1.648, 95% CI: 0.811–3.349, *P* = 0.167) (**Table 3**). There was no difference in the rates of mosaic blastocysts among the three groups.

**Table 3:** Comparison of preimplantation genetic testing for aneuploidy outcomes.

	GH co-treatment group (GH)	Self-control group (Non-GH)	*P* value^a^		Comparison group (Non-GH)		*P* value^b^
Number of enrolled patients	96	96			112		
Number of euploid blastocysts per patient	0.67 (0.97)	0.17 (0.45)	<0.01		0.36 (0.66)		0.020
Euploid blastocysts/Diagnosed blastocysts	64/200 (32.00%)	16/175 (9.14%)	<0.01		40/190 (21.05%)		0.015
Adjusted ORs (95% CI) *	4.765 (2.420–9.385)	reference	<0.01		—		—
Adjusted ORs (95% CI) **	1.930 (1.106–3.366)	—	—		reference		0.021
Mosaic blastocysts/Diagnosed blastocysts	36/200 (18.00%)	29/175 (16.57%)	0.715		30/190 (15.79%)		0.561
Adjusted ORs (95% CI) *	0.977 (0.537-1.776)	reference	0.938		—		—
Adjusted ORs (95% CI) **	1.134 (0.620-2.076)	—	—		reference		0.682
Patients with at least one euploid blastocyst/Patients	39/96 (40.63%)	13/96 (13.54%)	<0.01		31/112 (27.68%)		0.049
Adjusted ORs (95% CI) *	7.742 (2.950–19.926)	reference	<0.01		—		—
Adjusted ORs (95% CI)**	1.648 (0.811–3.349)	—	—		reference		0.167
Analysis of blastocyst euploidy rates according to age group							
<35 years							
Number of enrolled patients	16	16			15		
Euploid blastocysts/Diagnosed blastocysts	25/41 (60.98%)	4/34 (11.76%)	<0.01		12/42 (28.57%)		0.003
35-40 years							
Number of enrolled patients	40	40			49		
Euploid blastocysts/Diagnosed blastocysts	35/102 (34.31%)	5/65 (7.69%)	<0.01		20/93 (21.51%)		0.047
>40 years							
Number of enrolled patients	40	40			48		
Euploid blastocysts/Diagnosed blastocysts	4/57 (7.02%)	7/76 (9.21%)	0.757		8/55 (14.55%)		0.198

Data are presented as mean (standard deviation), N, or n/N (%).

a Comparison between GH cotreatment and self-control groups.

b Comparison between the GH co-treatment and comparison groups.

Comparisons between the GH co-treatment and self-control groups were performed using the Wilcoxon signed-rank test, McNemar’s test, Pearson’s chi-square test, or generalized estimating equations, as appropriate. Comparisons between the GH co-treatment and comparison groups were made using the non-parametric Mann–Whitney U test, generalized estimating equations, Pearson’s chi-square test, or logistic regression, as appropriate. Statistical significance was set at P < 0.05.

^*^Adjusted for total gonadotropin, oocytes, and ovarian stimulation protocol.

^**^Adjusted for age, anti-Müllerian hormone, body mass index, total gonadotropin, oocytes, infertility duration, infertility type, ovarian stimulation protocol, and the indication for preimplantation genetic testing for aneuploidy.

GH, growth hormone; OR, odds ratio; CI, confidence interval.

### Pregnancy outcomes of FET

Two patients in the GH co-treatment group and four in the comparison group insisted on the transfer of mosaic embryos. Ultimately, barring these patients, 80 euploid blastocysts (42 in the GH co-treatment and 38 in the comparison group) were transferred to 68 patients (39 in the GH co-treatment and 29 in the comparison group) in 79 transfer cycles (42 in the GH co-treatment and 37 in the comparison group). One couple in the comparison group insisted on the transfer of two euploid blastocysts, whereas for all patients in the other FET cycles, a single euploid blastocyst was transferred.

In FET cycles, no significant differences were observed in age, BMI, endometrial thickness, or endometrial preparation protocol. Compared to the comparison group, patients in the GH group had a significantly higher clinical pregnancy rate (73.81% vs 43.24%, OR: 4.538, 95% CI: 1.453–14.178, *P* = 0.009) (**Table 4**). There was no difference in the miscarriage rate per clinical pregnancy between the GH co-treatment and comparison groups (12.90% vs 25.00%, OR: 0.519, 95% CI: 0.113–2.391, *P* = 0.400). Compared with the comparison group, the live birth rate per embryo transfer procedure was higher in the GH co-treatment group (64.29% vs 32.43%, OR: 3.336, 95% CI: 1.472–7.560, *P* = 0.004) (**Table 4**). Live births occurred in 27 women (28.13%) in the GH co-treatment group and 12 women (10.71%) in the comparison group (OR, 3.564; 95% CI, 1.517–8.374; *P* =0.015) (**Table 4**).

**Table 4:** Comparison of pregnancy outcomes.

	GH co-treatment group (GH)	Comparison group (Non-GH)	*P* value
Number of patients with FET	39	29	
Number of embryo transfers	42	37	
Age (years)	36.19 (3.81)	36.73 (3.85)	0.534
BMI (kg/m^2^)	23.53 (2.26)	23.43 (3.33)	0.875
Endometrial thickness (cm)	0.93 (0.20)	0.89 (0.19)	0.271
Endometrial preparation protocol			
Natural ovulatory cycle	27/42 (64.29%)	16/37 (43.24%)	0.061
Artificial regimen	15/42 (35.71%)	21/37 (56.76%)	0.061
Implantation rate	31/42 (73.81%)	16/38 (42.11%)	0.004
Clinical pregnancy rate per embryo transfer	31/42 (73.81%)	16/37 (43.24%)	0.006
Adjusted ORs (95% CI) ^*^	4.538 (1.453–14.178)	reference	0.009
Miscarriage rate per clinical pregnancy	4/31 (12.90%)	4/16 (25.00%)	0.416
Adjusted ORs (95% CI) ^*^	0. 519 (0.113–2.391)	reference	0.400
live birth rate per embryo transfer	27/42 (64.29%)	12/37 (32.43%)	0.005
Adjusted ORs (95% CI) ^*^	3.336 (1.472–7.560)	reference	0.004
Live birth rate per group	27/96 (28.13%)	12/112 (10.71%)	0.001
Adjusted ORs (95% CI) ^**^	3.564 (1.517–8.374)	reference	0.004

Data are presented as mean (standard deviation), N, or n/N (%).

Both groups were followed until the first live birth or the first three euploid blastocyst transfers.

Comparisons of the live birth rate per group were made using binary logistic regression, and other comparisons were made using an independent sample t-test, Pearson’s chi-square test, and generalized estimating equations, as appropriate. Statistical significance was set at P < 0.05.

^*^Adjusted for age, BMI, endometrial thickness, endometrial preparation protocol, and indication for preimplantation genetic testing for aneuploidy.

^**^ Adjusted for age, BMI, the indication for PGT-A, and ovarian stimulation protocol.

GH, growth hormone; FET, frozen-embryo transfer; BMI, body mass index; NE, not estimated; OR, odds ratio; CI, confidence interval.

## Discussion

This study investigated the effect of GH supplementation on blastocyst aneuploidy in patients who underwent PGT-A cycle following failure. Our results present preliminary evidence that GH supplementation ameliorates blastocyst aneuploidy and improves clinical pregnancy and live birth rates in women who experienced prior pregnancy failures with no transferrable blastocysts, or the aneuploidy rate higher than 50% and no live birth was acquired, particularly in women younger than 40 years.

GH has been shown to have a consistent relationship with different parameters of embryonic quality, and higher concentrations of GH in follicular fluid are associated with rapid cleavage, good cleaving-embryo morphology, and high embryonic implantation potential (22). Further, other studies have shown that GH promotes oocyte maturation and embryonic development in cows (23), mice (24), and rhesus macaques (25). At our center, we had previously confirmed that GH promotes the maturation of human oocytes, presumably by accelerating the meiosis process, balancing the redox homeostasis of the cellular environment, and promoting the developmental competence of oocytes (26). A previous retrospective analysis reported that compared to the previous cycles, the number of biopsied and euploid embryos was significantly higher in a subsequent GH cycle, but the euploidy rate was not significantly different from the previous cycles (20). Indeed, our current study revealed an increasing trend in good-quality blastocysts and a significant increase in the number of euploid blastocysts. However, we also found that the euploidy rate was significantly higher in the GH co-treatment group than in the previous cycle and comparison groups. One plausible explanation for this is that GH improves the impaired separation of chromosomes associated with failed fertilization and poor early development potential of embryos in patients with high aneuploidy rates. This result is clinically relevant, as it increases opportunities for euploid embryo transfer in such patients and ultimately increases the probability of live birth.

In our study, there was a large range for age, so we conducted subgroup analysis, and there was no significant difference in the number of patients in each subgroup. However, in subgroup analysis, we found GH supplementation to significantly ameliorate the blastocyst euploidy rate among participants aged <35 and 35–40 years, but not among those older than 40 years, consistent with previous studies (27). As known, age diminishes the number of functional mitochondria, leading to impairments in chromosomal separation associated with failed fertilization. GH supplementation is known to improve mitochondrial activity, thereby directly and positively affecting the oocyte quality. In aged mice, GH supplementation administered before standard ovarian stimulation improves oocyte quality, likely by enhancing mitochondrial function (4). Administration of GH to older women upregulates the expression and activity of GHRs, which can improve mitochondrial function, oocyte quality, and fertilization rate (5). However, we did not find any improvement in aneuploidy rate in patients >40 years of age, this may be related to the fact that GH levels decrease significantly with increasing maternal age, and GH deficiency in patients over 40 years of age is more serious. In this study, approximately 42 days of low-dose (2 IU) GH supplementation was insufficient to improve GH deficiency. Further studies are required to determine whether a higher dosage of GH over a longer treatment period is required for such patients. In addition, interestingly, in our study GH supplementation improved aneuploidy rate in patients <35 years of age. Some previous studies have also found that exogenous GH can improve the quality of oocyte and enhance IVF outcomes in younger women with previous repeated IVF failures (15, 28).The possible explanation is that these younger women with high aneuploidy also lack growth hormone. However, the mechanism of exogenous GH improving aneuploidy in young women remains to be elucidated.

In addition, to reduce the limitation of using the preceding cycle as self-control, we added an additional comparison group. We found that the euploid rate of the comparison group was also higher, which might due to the adjustment of ovarian stimulation. Appropriate adjustments of ovarian stimulation protocol as well as type and dosage of gonadotropin were made for all women who failed previous PGT-A cycles because of no available embryos or high aneuploidy rate. This adjustment itself was supposed to lead to better prognosis, so the euploid rate of the comparison group might also increase. However, the euploid rate of the GH co-treatment group increased more significantly due to the effect of GH.

Regarding the effectiveness of GH supplementation in improving the live birth rate, the results of our study differ from many conclusions previously presented in the literature. For example, several randomized controlled trials did not detect any definitive benefits of GH administration on live birth rates in poor ovarian responders (29, 30). However, other studies have reported improvements in pregnancy, implantation, and live birth rates following GH supplementation (8, 10–12, 31). This discrepancy can be attributed to differences in patient groups. In this study, we observed more euploid blastocysts in the GH co-treatment group; in subsequent FET cycles, although all patients underwent euploid embryo transfer and without GH supplementation in the FET cycles, embryos from the GH co-treatment group were associated with higher clinical pregnancy and live birth rates. Growth hormones can improve the quality of oocytes and early embryonic development in a variety of ways, such as by improving mitochondrial function and regulating receptor expression. We speculated that this improvement may further improve long-term embryo developmental potential. Therefore, euploid embryos from GH-supplementation cycles had higher implantation and live birth rates than those from the comparison group; however, this hypothesis requires further investigation.

Regarding the effects of GH on the endometrium, a randomized controlled trial conducted in an oocyte-donation program to study the effects of GH on uterine receptivity in patients with repeated implantation failure showed that GH administration significantly increased endometrial thickness and the live birth rate (32). Moreover, in a prospective controlled study on the effects of GH on the clinical outcomes of frozen-thawed embryo transfer, Xue-Mei, et al. (33) found that the addition of GH significantly increased the clinical pregnancy, embryonic implantation, and live birth rates in addition to endometrial thickness, and serum vascular endothelial growth factor concentration. Another study showed that GH improved pregnancy outcomes in patients with thin endometria who underwent FET (34). In contrast, we did not observe any differences in endometrial thickness between the GH co-treated and comparison groups during either oocyte retrieval or FET cycles in our study. This difference may be due to the selection of patients and the decision to exclude patients with endometrial lesions. In addition, none of the patients received GH supplementation during the FET cycles. Therefore, we speculate that the increase in pregnancy rate may be due to the improved developmental potential of euploid embryos rather than the effects on endometrial thickness.

Although our findings are novel and inspiring, this study had some limitations. We included only 208 patients from our center in this study, and the sample size for subgroup analysis was relatively small, the universality of the results may thus be limited by the small sample size. The other limitation of the study lied in its non-interventional nature which lacked randomization, diverse reasons might make the participants to choose whether they accept the GH co-treatment or not. Considering the small sample size and mixed indications for PGT-A, further scientific research on the underlying mechanism and clinical trials with larger sample sizes are recommended to confirm the effects of GH and optimal protocols for its administration.

In conclusion, we present preliminary evidence showing that GH supplementation can ameliorate blastocyst aneuploidy and improve pregnancy outcomes in women younger than 40 years who have experienced prior pregnancy failures with aneuploidy rates higher than 50%. This evidence emphasizes the promise of clinical GH applications, especially for women who urgently need to improve their oocyte quality and euploidy rates, such as those who experienced previous PGT-A cycle failure due to an aneuploidy rate above 50%. This study also highlights the need for further scientific research on the mechanism underlying the effects of GH supplementation and clinical trials with larger sample sizes to confirm the conclusions and supplementation protocols.

## Data availability statement

In order to maintain the privacy of individuals participated in the study, the data underlying this article cannot be shared publicly.

## Author contributions

The study was conceived and designed by JY, Z-JC, and J-LM. Patients were recruited and selected by QG, MX, JJL, and JL. Data extraction and analysis were performed by PL, WZ, and QG. The initial draft of the manuscript was prepared by QG and subsequently revised by JY. All authors contributed to the article and approved the submitted version.
